# To a Green Economy across the European Union

**DOI:** 10.3390/ijerph191912427

**Published:** 2022-09-29

**Authors:** Romeo Victor Ionescu, Valentin Marian Antohi, Monica Laura Zlati, Lucian Puiu Georgescu, Catalina Iticescu

**Affiliations:** “Dunarea de Jos” University of Galati, 800008 Galati, Romania

**Keywords:** European policy, development, sustainability, green economy, innovation, econometrics

## Abstract

”Is the green economy a real solution to the present economic development?” represents the main question of the present research. The paper deals with assessing the impact of innovation on the green economy and quality of life through policies promoted at the European Union (EU) level. The objectives of the paper are to assess the impact level of the policy implementation across the Member States through the research and development (R&D) process and to identify models for the development of the green economy in Europe. The applied methods are empirical and analytical based on the study of literature, econometric modeling, pivoting econometric models, trend synthesis, prioritization, and staging of policy experimentation in the Member States through data processing and statistical programs. The results consist in obtaining development models for the green economy at the EU and national levels. In conclusion, the paper contributes to the early identification of vulnerabilities that may affect the development of European strategic projects in relation to events and security breaches occurring at the EU level at any given time.

## 1. Introduction

The green economy is characterized by the set of measures and procedures adopted by the Member States on the basis of a common agenda capable of improving the quality of life and the environment for European citizens.

The research question: Is the green economy a real solution to the present economic development? Based on the research question, we formulate the following research problem: The development of the European economy on a sustainable basis and in terms of the green economy can be a viable solution to overcome the current multi-crisis situation if and only if the level of development of the European core is homogenized in the context of technological progress, with the implementation of a viable plan to reduce regional disparities.

The aim of the research is to develop an applied econometric model based on the effective implementation of sustainable development solutions in the context of the Green Deal objectives.

The novelty of this approach is undeniable in the current geopolitical, economic, and energy context. Our paper aims to be a theoretical but practical tool, able to provide decision-makers with arguments in favour of supporting R&D-based sustainable development initiatives.

The main objective of this scientific approach is to quantify the policy implementation across the Member States through the research and development (R&D) process and to identify models for the development of the green economy in Europe.

The specific objectives of the research are the following:

O1. Identifying in the literature the main concepts, trends, and approaches related to sustainable development in the context of large-scale introduction of R&D in economic activity.

O2. Building a consolidated database of the evolution of the main indicators for monitoring the results achieved in the implementation of the Green Deal at the European level and the level of selected Member States.

O3. Defining the econometric model for quantifying development for the green economy at the EU and national levels.

O4. Testing, model validation, and proposals for practical solutions and policies to accelerate Green Deal development.

**Figure 1:**
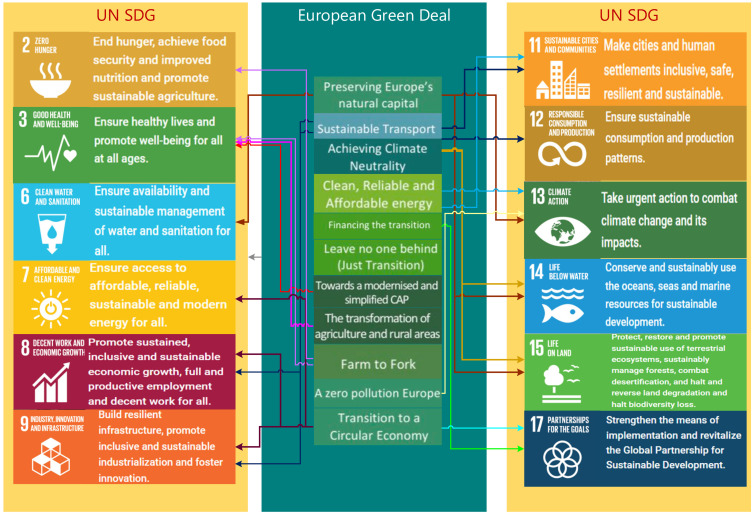
The link between the SDG targets and the EU green economy policy.

There are obvious links between the UN SGD and the Green Deal, which converge on the same courses of action and, in this context, support the approach we propose in this article (see Figure 1).

Green Deal—SDG convergence is a way to streamline the achievement of the targets, with bi-directional cost allocation and transparency of results; the convergence method also benefits from the complex databases that have been collected so far for the individual monitoring of sustainable development goals and Green Deal targets.

In order to substantiate a new model related to a green economy, we define the following working hypotheses:

**H1:**  *Economic development is a booster of R&D activity and an indirect instrument by which the objectives of the green economy and quality of life pursued at the EU level through specific policies and strategies, can be more easily achieved.*

**H2:**  *There are significant differences between the general European model and the national models, which demonstrates that the European proposed desideratum regarding sustainable development involves national disruptions when they are transferred through financing programs across the Member States’ economies.*

**H3:**  *The Member States with higher economic development always tend to approach faster and more faithfully the European model than those Member States with a developing economy.*

**H4:**  *The structural differences between the implementation of the sustainable development objectives across the Member States represent economic decelerators for the implementation of the policies at the European level.*

The article continues with Section 2, followed by Section 3, Section 4, and Section 6.

## 2. Theoretical Background

The common agenda contains 18 points, of which we have identified 5 major areas assimilated to the development of a European green economy, as follows sustainable development goals (SDG): SDG 6: ensuring universal access to safe and affordable drinking water, sanitation, and hygiene, and ending open defecation; SDG 7: ensuring universal access to modern energy services, improving energy efficiency and increasing the share of renewable energy; SDG 9: building resilient and sustainable infrastructure and promotes inclusive and sustainable industrialisation; SDG 11: to renew and plan cities and other human settlements in a way that offers opportunities for all; SDG 13: seeks to implement the commitment to the United Nations Framework Convention on Climate Change and deliver on the Green Climate Fund [1].

Regarding SDG6, clean water and sanitation, it aims to monitor and facilitate the access of the population to quality water sources with a direct impact on the health of the population and the environment over time. The connection with the economy is direct, as it is the major source of pollution. Areas such as industry and agriculture affect water resources by adding pollution to the production processes. During the last 15 years, the overall indicator has seen improvements at the European level. Still, the effect of monitoring the common agenda’s targets has allowed significant progress to be made on the key demons (part of our study), biochemical oxygen demand in rivers and phosphate in rivers. In the third monitored sector from the study, the results did not allow the calculation of the excellence indicators because the monitoring has been done for too short a time to evaluate nitrate in groundwater, and many statistical reports are missing at the Member State level [1].

Regarding the water resources, the big problem of the EU is the access to the water sources and the quality (unpolluted) water sources, as well. Access to water sources is impossible for 1.9% of the European population. This percentage has been reduced as a result of Member States’ efforts in recent years.

The water quality for rivers represented by biochemical oxygen demand (BOD) in European rivers has progressed from 2.7 mg O_2_·L^-1^ to 2.0 mg O_2_·L^-1^ during the last 15 years [2]. This aspect has contributed to the improvement of the quality of the EU hydrotechnical resources, an aspect that has also manifested itself at the demographic level by the increase in life expectancy.

Monitoring nutrient concentrations in surface and groundwaters is extremely important. The reduction of nutrient concentrations is one of the main objectives of the EU in terms of water quality.

Thus, from the data provided by Eurostat, pollution source, phosphates (PO^43-^), decreased significantly between 2002 and 2017, as they fell from 0.154 mg PO^43-^·L^-1^ in 2002 to a low of 0.093 mg PO^43-^·L^-1^ in 2017 [3]. This aspect is the result of the application of the provisions of the Urban Waste Water Treatment Directive for 25 years [4].

Nitrate concentrations (NO^3-^) are another important indicator in determining water quality. NO^3-^ it is in the nutrients category. This indicator was monitored in the groundwater and rivers. According to Eurostat data, the concentrations were relatively constant, with a slight upward trend: from 20.7 mg NO^3-^·L^-1^ (relatively constant values between 2003–2014), with a tendency to increase in the interval 2015–2020 up to 21.2 mg NO^3-^·L^-1^ [5]. It has been observed that from 1992 until now, the concentration of nitrates has been relatively constant, with a slight upward trend in recent years, but this could be attributed to the pluviometric deficit [6]. Nitrates come mainly from chemical fertilisers and are one of the main sources of surface and groundwater pollution.

Complex characterisation of the quality of watercourses is achieved, however, by calculating the water quality indices (WQI; WPI) that are determined by incorporating at least 10 physical-chemical parameters, and the concentrations of nitrates, phosphates, and BOD must be included [7,8].

The future directions for SDG6 monitoring of the common agenda are the conceptualisation of a Water Framework Directive, which aims to improve the water quality through a management strategy based on sustainable objectives [9]. According to this document, water resources have vital importance to the economy, the EU’s water-dependent sectors generating € 3.4 trillion, or 26% of the EU’s annual gross value added.

Regarding SDG 7, affordable and clean energy, it aims to ensure universal access to energy sources, as well as to increase renewable green energy contribution. This requires Member States’ steps to promote the investment in efficient and non-polluting energy sources. The future development directions are set out in strategies and policies for the general framework for climate change and energy monitoring [10]. The European Commission’s objectives in this domain are very ambitious for 2030: an increase to at least 32% share of renewable energy and 32.5% improvement in energy efficiency.

According to the proposed objectives regarding the clean energy agreement, significant results have been obtained during the last 15 years regarding the monitoring of energy consumption, reduction of energy consumption per household, monitoring and reduction of greenhouse gas emissions or the increase of green energy consumption. If the results are favourable for the last 15 years, it seems that the situation for some segments of interest has known a fundamental turn in the last 5 years. In relation to the common agenda’s proposed objectives, there has been insufficient progress in terms of primary energy consumption. In contrast, the dependence on the imported energy and final energy consumption has significantly exceeded the EU’s proposed objectives.

The only more accelerated trend in the last 15 years was found in terms of final energy consumption in households/capita, while energy production, greenhouse emissions and renewable energy maintained their upward trend in progress against the targets of the EU’s common development.

SDG9, Industry, innovation, and infrastructure, represents an area which has accumulated efforts to create a resilient and sustainable structure that promotes sustainable industrialisation based on research and innovation regarding the social, economic, and environmental challenges. For example, 2.19% of GDP was spent on R&D in the EU in 2018 [11]. There are sectors such as business, where R&D intensity increased to 1.45% of GDP in 2018. Higher education, government, and private administrations performed much more poorly. Another important indicator is the share of R&D personnel in the active population. It increased from 0.91% in 2003 to 1.35% in 2018 [11].

The development and innovation strategy is captured in an official document detailing the dynamics of innovation, including in terms of productivity and structural change, assessing investment in innovation and creating the concept of a general framework for innovation in Europe. The strategy covers strategic directions regarding the impact of renovation on the artificial intelligence applied to jobs, industrial dynamics in the presence of innovation, digital economy and scanning the innovative horizon [12].

SGD11, Sustainable cities and communities, aims to plan sustainable development at the level of cities and communities in order to ensure access to the sustainable public services and reduce their environmental impact. From the dynamics point of view, this field could be monitored only on four topics in the last fifteen years, aimed at air pollution (in moderate progress), the rate of deaths in car accidents (in moderate progress), the recycling rate of community waste (in significant progress) and the share of buses and trains in total passenger transport (in insufficient progress). In the short term, in the last five years, most objectives (6 of 10) have seen significant progress. On the other hand, there are two indicators that have made insufficient progress: people killed in road accidents and the share of buses and trains in total passenger transport. Moreover, the settlement area per capita indicator has declined compared with the EU target.

Regarding the waste, 47.4% of the total community waste generated in the EU was recycled in 2018. Against this level, the EU target is to recycle 60% of EU waste in 2030. The future EU strategy is contained in the report of the commission on Air quality in Europe 2020 [13]. In this document, waste is seen as the main generator of CH4 emissions.

SGD13: Climate Action aims to adhere to the general framework of the UN Convention on Climate Change. The aim of this is to strengthen national resilience and to improve the adaptive capacity to climate change and natural disasters. The EU succeeded in reducing greenhouse emissions by 16.2% during 2003–2018 and proposed to achieve net-zero GHG emissions by 2050 [14].

A green European economy is an important desideratum for the EU’s further development. This desideratum is also supported by the ambitious goal of eliminating pollution by 2050 and increasing quality of life, corroborated with the efforts for sustainable development and reducing the impact of the economy on the environment.

Important indicators of this strategy aimed at the objectives of the medium and long-term European Agenda on the environment, sustainable economic development, energy security and safety, etc.

These approaches are widely debated in the literature. Thus, regarding the need for water quality, we have identified approximately 17,500,000 specialised works worldwide in the last ten years, especially focused on: river water quality, water quality parameters, drinking water quality, lake water quality, aquaculture water quality, reservoir water quality, irrigation water quality, and macroinvertebrates water quality. It results that, besides the global character of the research, the field represents a pole of interest for global actuality; the authors of this paper note the scientific approach regarding the application of the neural models in the matter of water quality (BOD) [15].

An interesting analysis realised by Wang et al. [16] is based on a mix of models (SSU and PP model, spatial analysis method, spatial econometric model). It puts into connection people’s welfare and green economy efficiency. The main conclusion of this approach is that green economy efficiency is much harder to achieve and is lower than conventional efficiency, with the main support of green economy efficiency being the tertiary sector of the economy.

Modelling green economy efficiency with the help of the dynamic spatial panel Durbin model and mediating effect model is realised by Yuan et al. [17]. The basic idea is that the development of a green economy needs an improvement in green economic efficiency. The authors believe that in order to improve environmental economic efficiency, it is necessary to dynamically adjust regional policies related to the transformation and modernisation of the industrial structure.

Other authors [18] considered that it is appropriate to apply linear multiple regression models for BOD removal efficiency of different sewage treatment technologies. These authors present three different sewage treatment technologies: Activated Sludge Process (ASP), Extended Aeration, and Densadeck. According to the United Nations there are three traditional dimensions of sustainable development: economic, social, and environmental. A fourth institutional dimension (governance) has also been included. The EU has developed its own dashboard, which covers 100 indicators. The authors used the correlations between these indicators grouped into three major components: economic and social indicators: income/poverty, health, education/employment, and governance. The result of the analysis points out that the Western and Northern Europe countries have a better position than the East and Southern Europe countries, which is supported by their superior development level [19].

Another factor with an impact on the environment is nitrates. According to some experts [20], agriculture represents a segment with major potential for damage. This results from the joint analysis of the EU Sustainable Development Goal (SDG), Common Agricultural Policy (CAP) and Agri-Environmental Indicator (AEI). Regarding Objective 1 (No poverty), SDG affects the people at risk of poverty or social exclusion. In turn, CAP affects the share of the population at risk of poverty or social exclusion in thinly populated areas. Objective 6 (Clean water and sanitation) is affected by the level of nitrates in the groundwater (under the impact of SDG) and by the level of nitrates in the freshwater (groundwater quality under the impact of CAP and the impact of the AEI).

Some authors show an interest in renewable energy consumption. They perform “scenarios” for energy consumption forecasts in the EU of 2020 (Renewable Energy in Final Energy Consumption and Income in the EU Countries). According to them, the UK should have been part of cluster 1 of the Share of Renewable Energy in Electricity and GDP per capita with a calculated share of 9.6% in 2020 [21].

Instead, other authors [22] appreciate that there is a strong synergy between the objective of SDG 7 (affordable and clean energy) and SDG 8 (decent work and economic growth). On the other hand, SDG 12 (responsible production and consumption) accounts for most of the future renewable electricity price variation.

A distinct and interesting approach is that of sustainable demand-supply balance for copper in the long term. The authors evaluate the copper waste industry by applying trend markers based on the operating conditions induced by the environmental policy promoted globally, with the change of the resilience and fairness curve and by marking a downward trend of security operations [23].

The authors of a study conducted following the onset of the pandemic, which aims at the evolution of GDP and CO_2_ equivalent emissions, compared the Business-as-usual (BAU) policy and the corresponding situation to the pre-COVID-19 era. They show that the economic contraction had a positive effect on limiting the CO_2_ emissions, so that a 10% decrease in the level of activity leads to a 7% increase in the recession (decrease in GDP) and a 5% limitation of the CO_2_ emissions, in the conditions of maintaining the demand (variation 0.2%) [24].

Another facet of the global issue of sustainability and environmental quality is waste management. This dimension has been integrated into a concept whose implementation at the EU level i an increasingly watched, smart city, thus being placed under the jurisdiction of local authorities, benefiting from financing and development projects increasingly managed and timely [25].

Some authors, such as Chenggang Li and Yong Gan [26], focus their research on the role of finance in promoting green development. By using the spatial Dubin model based on the distance weight matrix and empirical analyses, the authors conclude that the growth of green finance supports the quality of the regional environment, as it manifests itself as a significant positive spatial spillover effect.

The green economy efficiency can become a support for urban green economy in developing economies in the approach of Yajie Liu and Feng Dong [27]. The main condition of this process is the implementation of technological innovation. The analysis covers almost 300 prefecture cities from Chine. The authors use a game cross-efficiency model and a spatial econometric model in order to connect technological innovation and green economy efficiency in using natural resources during urbanisation. The solution proposed by the authors consists of building a green economy city cluster to achieve regionally common development.

A special approach is taken by Odugbesan et al. [28], who consider the implications of financial regulations on the sustainable green economy in Turkey. The analysis covers a significant statistical period (1996–2019). It is based on Perron and Lee-Strazicich unit root test in the presence of a structural break point for examining the stationarity properties of the series. Moreover, the authors introduce FMOLS, CCR, and ARDL for estimating the long and short-run effects of financial regulations on carbon productivity. The main result of the study is that there is a long-run causal relationship between law, economic freedom, inflation and carbon productivity.

According to Junwei Ma et al. [29], green economic efficiency can be used as an index to measure economic, social, and environmental development. The analysis focuses on the quantification of two indicators: static green economic efficiency and dynamic green total factor productivity. In order to achieve this, the authors use slack-based measurement (SBM) directional distance function and Luenberger productivity indicator under conditions of uncertainty. The main conclusion of the analysis is the highlighting of the positive correlation between the two above-mentioned indicators.

Green economic growth is analysed by Qian et al. [30] in connection with financial and economic development. The analysis is focused on two elements: growth promotion and energy conservation/emission reduction, and uses Slacks Based Model-Data Envelopment Analysis (SBM-DEA). The authors point out that financial agglomeration has a great positive effect on green economic growth.

An interesting opinion is that the pandemic was an incentive for green development by Chai et al. [31]. The authors use models such as slacks-based measure (SBM) and Malmquist–Luenberger (ML) index in order to quantify green total factor productivity and how it can be influenced by FDI. The research is intended to support policymakers in introducing FDI of high quality at the regional level for green economic development.

The positive impact of education and R&D spending on sustainable development is analysed by Zhang et al. [32] by using as research methods generalised method of moments (GMM) and data envelopment analysis (DEA). The research is focused on the following aspects: public spending on R&D, green economic growth, and energy efficiency. The study concludes that regional disparities in economic development lead to different investments and impacts in terms of investments in new green technologies.

The impact of fintech and green finance on green growth was analysed by Zhou et al. [33]. The result of the research was the definition of an index to evaluate the green growth of the regional economy based on the in-depth analysis of the influence mechanism of green finance on green growth. This index is able to explain regional disparities in economic development through the contribution that green finance makes to development.

The entire previous analysis motivates our scientific approach, whose purpose is to improve the current conditions by enhancing the innovative approach based on R&D&I activity as an operational link of the objectives of quality of life, environment, and sustainable economy in the EU.

## 3. Methodology

**Figure 2:**
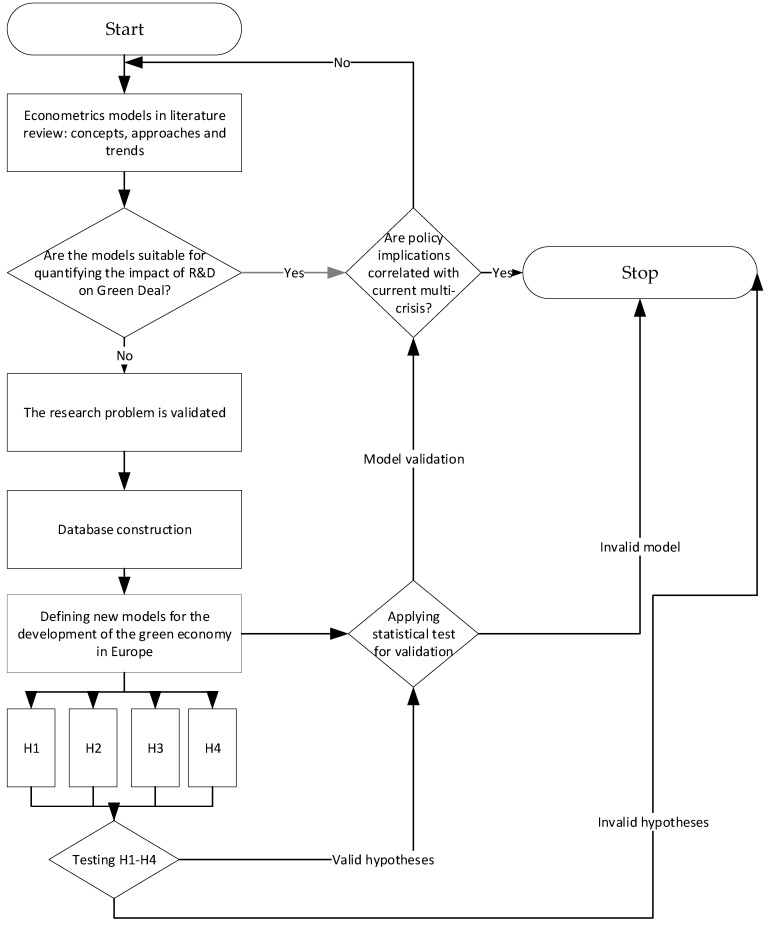
Diagram of the research procedure.

The study was carried out according to the following research chart (see Figure 2).

As a result of the aspects presented in the Theoretical Background, we appreciate that it is necessary to make a correlation of the contribution of innovation research to the improvement of these sectors as an operational binder transition to the green economy in 4 Member States depending on their economic development, as follows: Germany, Ireland, Romania and Bulgaria.

These countries were selected on the basis of their share of R&D expenditure in GDP, the first two having high shares, while the last two have low shares of this indicator in GDP.

The model is based on the structured evolution in dynamics of the indicators presented in correlation with the R&D dynamics in the analysed Member States. The analysis was performed on two distinct levels to identify the current trend, respectively a trend analysis in relation to point 0, the year 2000, and to identify the annual trend in relation to the average evolution of the indicators during the last 25 years.

The analysis is based on an initial raw statistical database [2,3,6,9,10,11,12,13,14]. This database has been consolidated and revised in order to achieve comparability of indicators using the moving average method.

We applied the linear regression model (least squares method) in order to evaluate the European impact profile of R&D on the green economy’s development and the citizens’ quality of life. The results of the model aimed at evaluating the regression variables on air quality (“*oxi*”), the level of greenhouse gas emissions (“*greenhouse*”), water quality (“*phosriv*”), environmental quality through the size of greening and reducing the impact of pollution due to good management of waste (“*waste*”), the energy dimension—renewable component (“*renew*”) and, last but not least, the size of the population involved in R&D (“*r & dpers*”). The regression variables were analysed in relation to the dependent variable, R&D progress at the regional level (“*r & d*”).

The regression function has the following shape:

(1) $$\hat{r\&d_{i}}=\alpha_{1}{}_{i}\cdot oxi_{i}+\alpha_{2}{}_{i}\cdot phosriv_{i}+\alpha_{3}{}_{i}\cdot renew_{i}+\alpha_{4}{}_{i}\cdot greenhouse_{i}+\alpha_{5}{}_{i}\cdot waste_{i}+\alpha_{6}{}_{i}\cdot r\&dpers_{i}+\varepsilon_{i}$$

where: $\hat{r\&d_{i}}$—dependent variable; *i*—the region for which the model is calculated; $\alpha_{1}$–$\alpha_{6}$—regression coefficients; ε—residual value.

Applying the model to the collected database allows to define the following regional system of equations:

(2) $$\left\{ \begin{array}{c} \hat{r\&dEU}=-0.067oxiEU+1.148phosrivEU+0renewEU-0.019greenhouseEU-0.002wasteEU+0.317r\&dpersEU+3.413\\ \hat{r\&dBG}=0.024oxiBG-0.404phosrivBG+0.021renewBG-0.005greenhouseBG-0.004wasteBG+0.405r\&dpersBG+0.46\\ \hat{r\&dIR}=0.069oxiIR+16.808phosrivIR-0.131renewIR-0.026greenhouseIR+0.039wasteIR+0.48r\&dpersIR+2.684\\ \hat{r\&dF}=-0.036oxiF-1.474phosrivF+0.015renewF-0.013greenhouseF-0.013wasteF-0.243r\&dpersF+4.137\\ \hat{r\&dRO}=-0.081oxiRO+0.445phosrivRO+0.013renewRO+0.009greenhouseRO+0.004wasteRO-0.668r\&dpersRO+0.148 \end{array}\right.$$

Testing the level of homogeneity of green economy development is done according to the formula:

(3) $$Skewness_{i}=\frac{\sum_{j=1}^{5}{\left(\alpha_{ij}-\bar{\alpha_{i}}\right)}^{3}}{\left(j-1\right)\cdot \sqrt{\frac{\sum_{j=1}^{5}{\left(\alpha_{ij}-\bar{\alpha_{i}}\right)}^{2}}{j}}}$$

where $\alpha_{ij}$—regional centres of disparity calculated from the Equation (2); $\bar{\alpha_{i}}$—average of the regional disparity centre for each indicator analysed; *j*—the number of model regression variations (Equation (1)).

**Figure 3:**
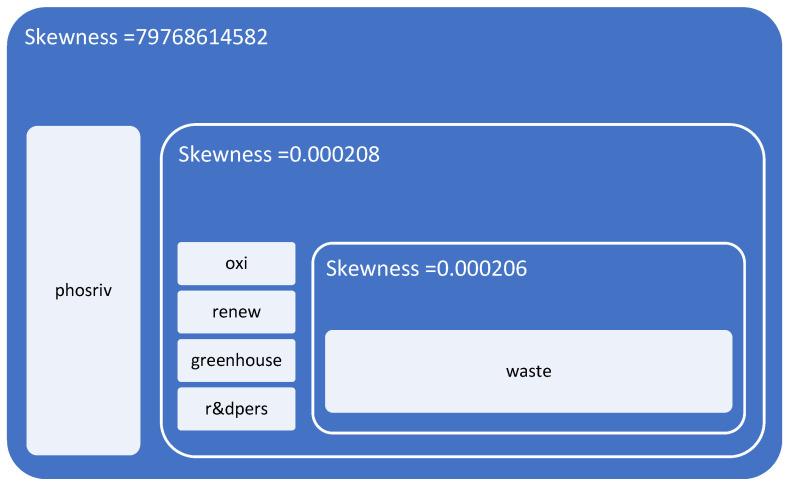
Green economy disparities in the EU.

The obtained value of the function reveals, on the basis of the model coefficients in Equation (2), the following distribution of disparities at the regional level for the studied states (see Figure 3).

From the application of the model, a cohesion of waste management policies (“*waste*”) results as a fundamental element in the construction of the European green economy in relation to allocated R&D expenditure. Secondly, significant cohesion was determined for air quality (“*oxi*”), the level of greenhouse gas emissions (“*greenhouse*”), the energy dimension—renewable component (“*renew*”) and the size of the population involved in R&D (“*r & dpers*”). The biggest disparities assessed at the European level are those related to water quality (“*phosriv*”), an area that affects, on the one hand, the quality of drinking water and the health of consumers and on the other, the quality of aquatic fauna and therefore the health of European consumers.

At the regional level, structural differences in economies (industrialised economies, consumer economies) significantly influence water quality. We presume that smart water management policy should be approached according to regional specificities.

## 4. Results

The database used for modelling was represented by the statistics for the last 20 years (2000–2020) of the model indicators for the European dimension. The model is presented in Table 1.

The representativeness of the general European model is 98.3%, which gives a high statistical significance. It is completed by the small number of degrees of freedom of the regression variables (6) compared with the total of 20 degrees of freedom of the model.

The ANOVA test for the European model reflects a low level of representation of the residual variable and a Sig coefficient tending to 0, which validates the model by rejecting the null hypothesis and validating the alternative hypothesis (see Table 2).

**Figure 4:**
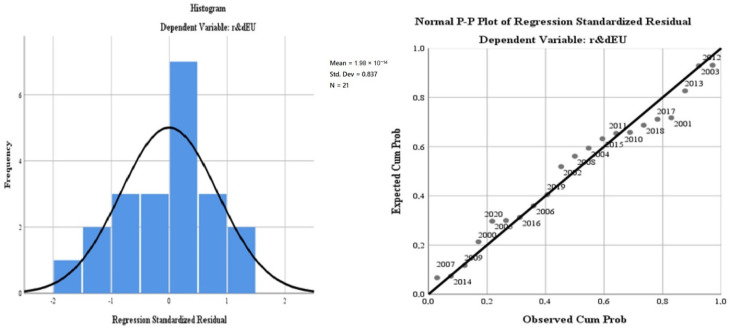
Regression analysis for the EU27 during 2000–2020. Source: Data operated under IBM-SPSS 25 (IBM, Armonk, NY, USA).

The results of the regression analysis are presented in Figure 4. The test of standardised coefficients shows that:

- The variation of the regressor regarding the air quality (“*oxiEU*”) in relation to the dependent variable reflects an inversely proportional correlation of −6.7%;
- The variation of the regressor regarding water quality (“*phosrivEU*”) in relation to the dependent variable, reflects a directly proportional correlation of 114.8%;
- The variation of the regressor regarding the energy dimension—renewable component (“*renewEU*”) in relation to the dependent variable reflects a directly proportional correlation of 0%;
- The variation of the regressor regarding the level of greenhouse gas emissions (“*greenhouseEU*”) in relation to the dependent variable reflects an inversely proportional correlation of −1.9%;
- The variation of the regressor regarding the quality of the environment through the dimension of greening and reducing the impact of pollution as a result of good waste management (“*wasteEU*”), in relation to the dependent variable, reflects an inversely proportional correlation of −0.2%;
- The variation of the regressor regarding the size of the population involved in R&D (“*r & dpersEU*”) in relation to the dependent variable reflects a directly proportional correlation of 31.7%.

Under the second step of the analysis, we applied the linear regression model (least squares method) to evaluate the Bulgarian impact profile of R&D on the green economy’s development and the citizens’ quality of life.

The results of the model aimed at evaluating the regression variables on air quality (“*oxiBG*”), the level of greenhouse gas emissions (“*greenhouseBG*”), water quality (“*phosrivBG*”), environmental quality through the size of greening and reducing the impact of pollution due to good management of waste (“*wasteBG*”), the energy dimension—renewable component (“*renewBG*”) and, last but not least, the size of the population involved in R&D (“*r & dpersBG*”). The regression variables were analysed in relation to the dependent variable, R&D progress in Bulgaria (“*r & dBG*”). The database used for modelling was represented by the statistics for the last 20 years (2000–2020) of the model indicators for the Bulgarian dimension. The model is presented in Table 3.

The representativeness of the Bulgarian model is 80.1% (lower than the general European model), which gives a high level of statistical significance. It is completed by the small number of degrees of freedom of the regression variables (6) compared with the total of 20 degrees of freedom of the model.

The ANOVA test for the European model reflects a low level of representation of the residual variable and a Sig coefficient tending to 0, which validates the model by rejecting the null hypothesis and validating the alternative hypothesis (see Table 4).

**Figure 5:**
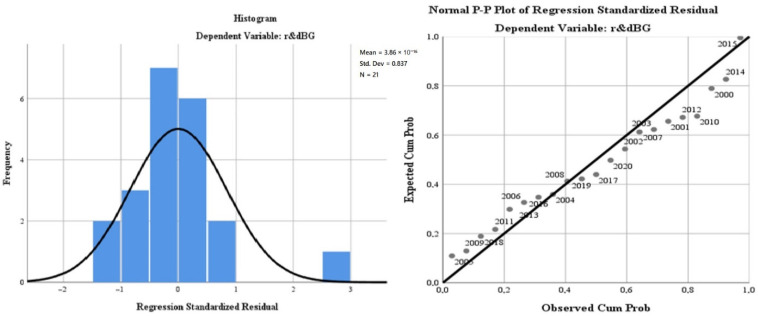
Regression analysis for Bulgaria during 2000–2020. Source: Data operated under IBM-SPSS 25 (IBM, Armonk, NY, USA).

The results of the regression analysis are presented in Figure 5.

The test of standardised coefficients shows that:

- The variation of the regressor regarding the air quality (”*oxiBG*”) in relation to the dependent variable reflects a directly proportional correlation of 2.4%;
- The variation of the regressor regarding water quality (”*phosrivBG*”) in relation to the dependent variable, reflects an inversely proportional correlation of −40.4%;
- The variation of the regressor regarding the energy dimension—renewable component (”*renewBG*”) in relation to the dependent variable reflects a directly proportional correlation of 2.1%;
- The variation of the regressor regarding the level of greenhouse gas emissions (“*greenhouseBG*”) in relation to the dependent variable, reflects an inversely proportional correlation of −0.5%.

Using the third step of the analysis, we applied the linear regression model (least squares method) to evaluate the Irish impact profile of R&D on the green economy’s development and the citizens’ quality of life.

The results of the model aimed at evaluating the regression variables on air quality (“*oxiIR*”), the level of greenhouse gas emissions (“*greenhouseIR*”), water quality (“*phosrivIR*”), environmental quality through the size of greening and reducing the impact of pollution due to good management of waste (“*wasteIR*”), the energy dimension—renewable component (“*renewIR*”) and, last but not least, the size of the population involved in R&D (“*r & dpersIR*”). The regression variables were analysed in relation to the dependent variable, R&D progress in Ireland (“*r & dIR*”). The database used for modelling was represented by the statistics for the last 20 years (2000–2020) of the model indicators for the Irish dimension. The model is presented in Table 5. We applied the linear regression model (least squares method) in order to evaluate the Irish impact profile of R&D on the green economy’s development and the citizens’ quality of life. The results of the model aimed at evaluating the regression variables on air quality (“*oxiIR*”), the level of greenhouse gas emissions (“*greenhouseIR*”), water quality (“*phosrivIR*”), environmental quality through the size of greening and reducing the impact of pollution due to good management of waste (“*wasteIR*”), the energy dimension—renewable component (“*renewIR*”) and, last but not least, the size of the population involved in R&D (“*r & dpersIR*”). The regression variables were analysed in relation to the dependent variable, R&D progress in Ireland (“*r & dIR*”). The database used for modelling was represented by the statistics for the last 20 years (2000–2020) of the model indicators for the Irish dimension. The model is presented in Table 5.

The representativeness of the Irish model is 65.3% (lower than the general European model), which gives a medium level of statistical significance. It is completed by the small number of degrees of freedom of the regression variables (6) compared with the total of 20 degrees of freedom of the model.

The ANOVA test for the European model reflects a low level of representation of the residual variable and a Sig coefficient tending to 0, which validates the model by rejecting the null hypothesis and validating the alternative hypothesis (see Table 6).

**Figure 6:**
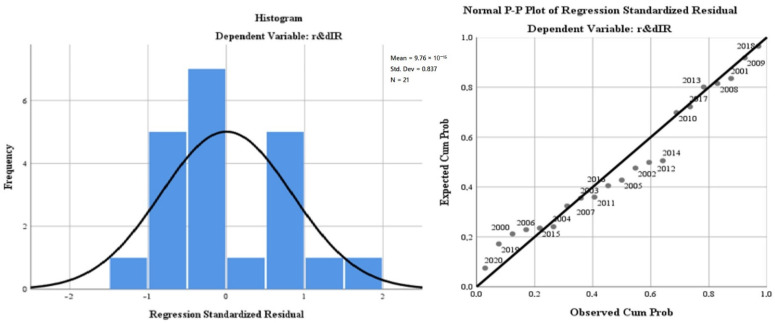
Regression analysis for Ireland during 2000–2020. Source: Data operated under IBM-SPSS 25 (IBM, Armonk, NY, USA).

The results of the regression analysis are presented in Figure 6.

The test of the standardised coefficients shows that:

- The variation of the regressor regarding the air quality (“*oxiIR*”) in relation to the dependent variable reflects a directly proportional correlation of 6.9%;
- The variation of the regressor regarding water quality (“*phosrivIR*”) in relation to the dependent variable, reflects a directly proportional correlation of 1680.8%;
- The variation of the regressor regarding the energy dimension—renewable component (“*renewIR*”) in relation to the dependent variable reflects an inversely proportional correlation of −13.1%;
- The variation of the regressor regarding the level of greenhouse gas emissions (“*greenhouseIR*”) in relation to the dependent variable reflects an inversely proportional correlation of −2.6%;
- The variation of the regressor regarding the quality of the environment through the dimension of greening and reducing the impact of pollution as a result of good waste management (“*wasteIR*”), in relation to the dependent variable, reflects a directly proportional correlation of 3.9%;
- The variation of the regressor regarding the size of the population involved in R&D (“*r & dpersIR*”) in relation to the dependent variable reflects a directly proportional correlation of 48%.

For the fourth time, we applied the linear regression model (least squares method) to evaluate the French R&D impact profile on the green economy’s development and the citizens’ quality of life. The results of the model aimed at evaluating the regression variables on air quality (“*oxiF*”), the level of greenhouse gas emissions (“*greenhouseF*”), water quality (“*phosrivF*”), environmental quality through the size of greening and reducing the impact of pollution due to good management of waste (“*wasteF*”), the energy dimension—renewable component (“*renewF*”) and, last but not least, the size of the population involved in R&D (“*r & dpersF*”). The regression variables were analysed in relation to the dependent variable, R&D progress in France (“*r & dF*”). The database used for modelling was represented by the statistics for the last 20 years (2000–2020) model indicators for the French dimension. The model is presented in Table 7.

The representativeness of the French model is 58.1% (lower than the general European model), which gives a medium level of statistical significance. It is completed by the small number of degrees of freedom of the regression variables (6) compared with the total of 20 degrees of freedom of the model.

The ANOVA test for the French model reflects a low level of representation of the residual variable and a Sig coefficient tending to 0, which validates the model by rejecting the null hypothesis and validating the alternative hypothesis (see Table 8).

**Figure 7:**
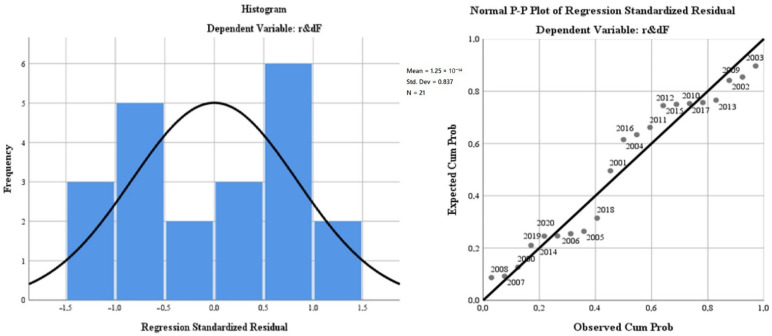
Regression analysis for France during 2000–2020. Source: Data operated under IBM-SPSS 25 (IBM, Armonk, NY, USA).

The results of the regression analysis are presented in Figure 7.

The test of the standardised coefficients shows that:

- The variation of the regressor regarding the air quality (“*oxiF*”) in relation to the dependent variable reflects an inversely proportional correlation of −3.6%;
- The variation of the regressor regarding water quality (“*phosrivF*”) in relation to the dependent variable, reflects an inversely proportional correlation of −147400%;
- The variation of the regressor regarding the energy dimension—renewable component (“*renewF*”) in relation to the dependent variable reflects a directly proportional correlation of 1.5%;
- The variation of the regressor regarding the level of greenhouse gas emissions (“*greenhouseF*”) in relation to the dependent variable reflects an inversely proportional correlation of −1.3%;
- The variation of the regressor regarding the quality of the environment through the dimension of greening and reducing the impact of pollution as a result of good waste management (“*wasteF*”), in relation to the dependent variable, reflects an inversely proportional correlation of −1.3%;
- The variation of the regressor regarding the size of the population involved in R&D (“*r & dpersF*”) in relation to the dependent variable reflects an inversely proportional correlation of −24.3%.

Finally, we applied the linear regression model (least squares method) to evaluate the Romanian R&D impact profile on the green economy’s development and the citizens’ quality of life. The results of the model aimed at evaluating the regression variables on air quality (“*oxiRO*”), the level of greenhouse gas emissions (“*greenhouseRO*”), water quality (“*phosrivRO*”), environmental quality through the size of greening and reducing the impact of pollution due to good management of waste (“*wasteRO*”), the energy dimension—renewable component (“*renewRO*”) and, last but not least, the size of the population involved in R&D (“*r & dpersRO*”). The regression variables were analysed in relation to the dependent variable, R&D progress in Romania (“*r & dRO*”). The database used for modelling was represented by the statistics for the last 20 years (2000–2020) of the model indicators for the Romanian dimension. The model is presented in Table 9.

The representativeness of the Romanian model is 33.6% (minimum compared with the general European model), which gives a minimum level of statistical significance. It is completed by the small number of degrees of freedom of the regression variables (6) compared with the total of 20 degrees of freedom of the model.

The ANOVA test for the Romanian model reflects a low level of representation of the residual variable and a Sig coefficient tending to 0, which validates the model by rejecting the null hypothesis and validating the alternative hypothesis (see Table 10).

**Figure 8:**
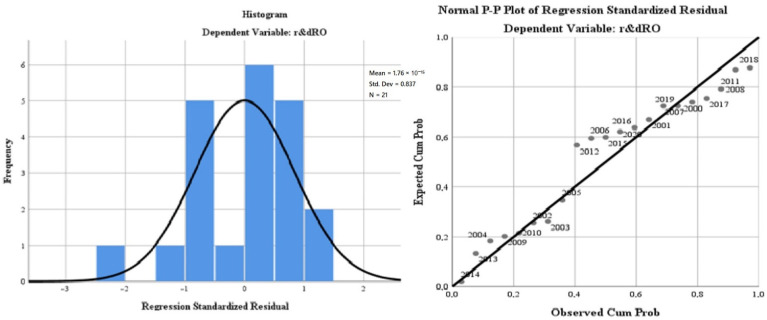
Regression analysis for Romania. Source: Data operated under IBM-SPSS 25 (IBM, Armonk, NY, USA).

The results of the regression analysis are presented in Figure 8.

The test of the standardised coefficients shows that:

- The variation of the regressor regarding the air quality (“*oxiRO*”) in relation to the dependent variable reflects an inversely proportional correlation of −8.1%;
- The variation of the regressor regarding water quality (“*phosrivRO*”) in relation to the dependent variable, reflects a directly proportional correlation of 44.5%;
- The variation of the regressor regarding the energy dimension—renewable component (“*renewRO*”) in relation to the dependent variable reflects a directly proportional correlation of 1.3%;
- The variation of the regressor regarding the level of greenhouse gas emissions (“*greenhouseRO*”) in relation to the dependent variable reflects a directly proportional correlation of 0.9%;
- The variation of the regressor regarding the quality of the environment through the dimension of greening and reducing the impact of pollution as a result of good waste management (“*wasteRO*”), in relation to the dependent variable, reflects a directly proportional correlation of 0.4%;
- The variation of the regressor regarding the size of the population involved in R&D (“*r & dpersRO*”) in relation to the dependent variable reflects an inversely proportional correlation of −66.8%.

## 5. Discussions

**Figure 9:**
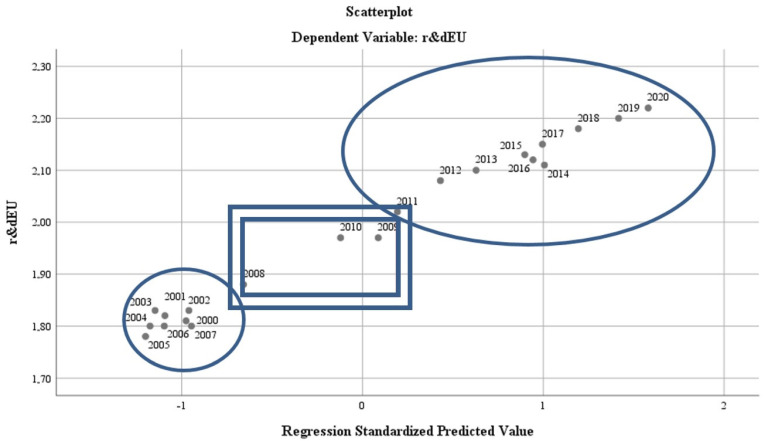
Regression analysis on two levels. Source: Data operated under IBM-SPSS 25 (IBM, Armonk, NY, USA).

The results of the model reflect the fact that there are quantifiable results of the impact of the application of green policies on the EU economy and quality of life. This conclusion is confirmed by the significant results in terms of homogeneity and statistical significance of the general model, which presents a bipolarity of the results on two-time levels. The first level is concentrated on the period 2000–2007, while the second level on the period 2010–2020. The economic crisis of 2008–2010 marks the frontier for the efficient implementation of the measures (see Figure 9).

**Figure 10:**
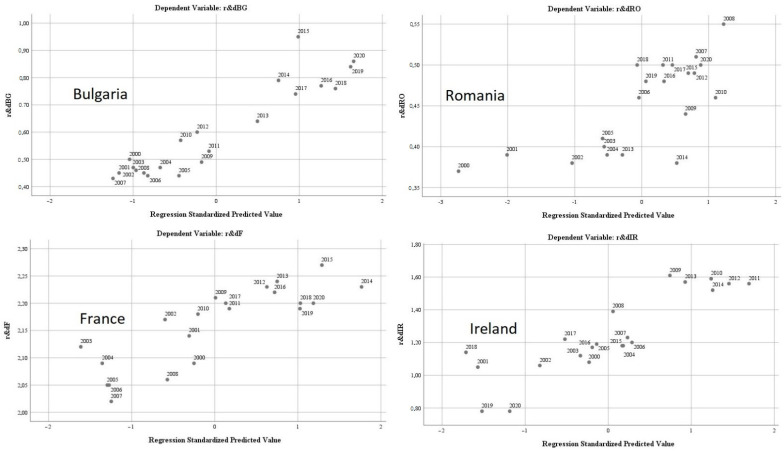
Regression analysis on analysed four countries vs the average EU situation during 2000–2020. Source: Data operated under IBM-SPSS 25 (IBM, Armonk, NY, USA).

At the European level, the main syncope in supporting the strategic objectives on green policies and quality of life is reflected during the economic crisis. There are significant differences in the impact of syncopes in terms of strategies at the national level. Regarding the three of the four studied cases (Bulgaria, France, and Ireland), the effect of the Markov chains on the national economies after the manifestation of syncopes in the general profile is noticeable in the sense that the syncopes last at least a few years after the manifestation at the general profile or take over elements of cyclicality (the French economy). On the other hand, in the case of Romania, it is observed that the syncopes have nothing to do with those generated at the level of the general profile and manifested themselves in the pre-accession period (2003–2006) when Romania approached some European objectives in terms of green economy and quality of life. The fact that the changes compared with the general profile are significant indicates the weak adherence to the sustainable development objectives considered by the EU (see Figure 10).

This argument, corroborated with the obtained results from the modelling, proves the hypotheses H1 and H2.

**Figure 11:**
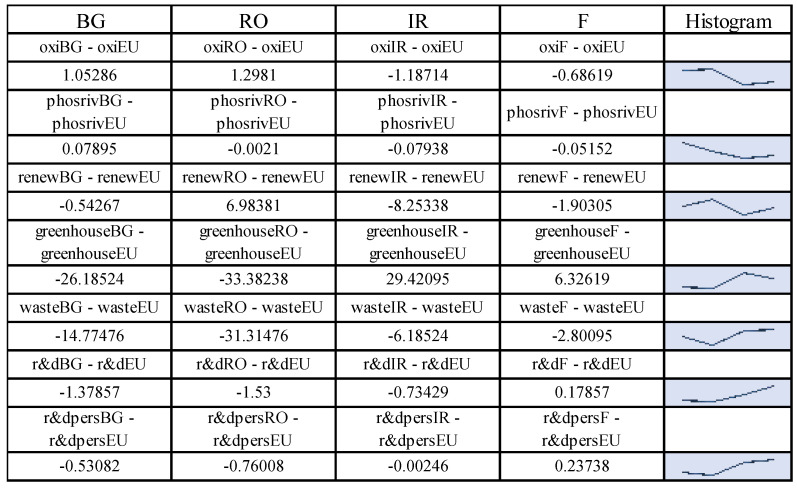
Comparative analysis between the analysed economic entities. Source: Data operated under IBM-SPSS 25 (IBM, Armonk, NY, USA).

In order to demonstrate the H3 hypothesis, we proceeded to analyse by data pairs the frequency series of regression variables segregated by country of origin, and we observed that, at the level of the differences between the data pairs with the general profile, the results show the proximity or distance of the objectives of the common policy depending on the level of development of the analysed state (see Figure 11).

According to Figure 11, it is found that the values closest to the general profile are obtained at the average level for France (1.3 points compared with the general profile), while Ireland is at a positive distance of 12.97 points from the general profile with a more advanced green economy. Bulgaria has a negative gap of 42 points compared with the general profile, and Romania has a negative gap of 58 points compared with the general profile. These results were obtained by summing the point differences on the indicator. This result demonstrates the H3 hypothesis. 

**Figure 12:**
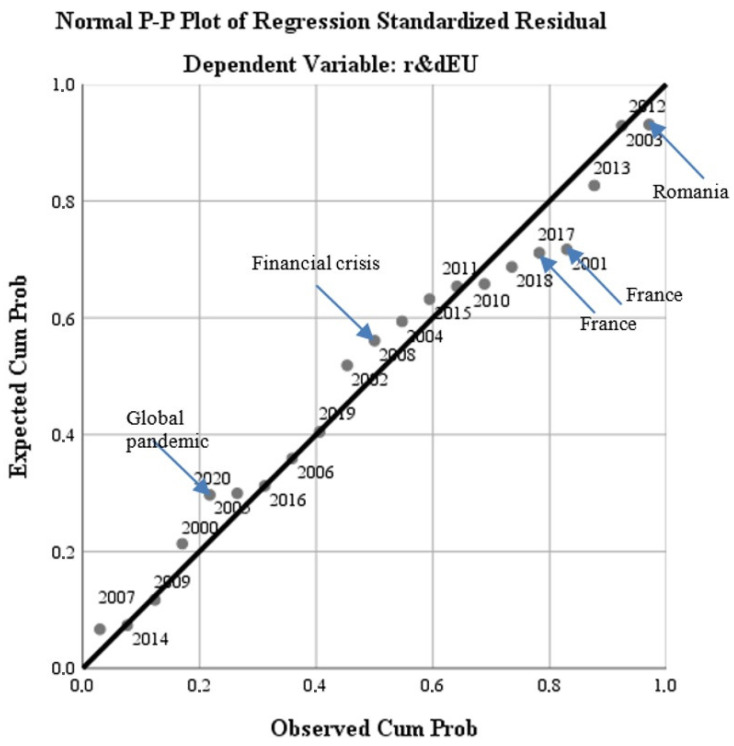
Variations/inflections of the normal distribution from the predicted line during 2000–2020. Source: Data operated under IBM-SPSS 25 (IBM, Armonk, NY, USA).

From the distribution diagram of the frequencies of the dependent variable in the general profile, using the annual markers (time series), it is observed that the variations/inflections of the normal distribution to the predicted right coincide in some moments with the events produced at Member States (France) or in accession course (Romania in the pre-accession phase), (see Figure 12).

This evolution demonstrates the H4 hypothesis. 

On the basis of the study hypotheses validated above, the following policies applicable at the level of the Member States analysed and at the EU level in extenso can be drawn (see Table 11):

This connection between green economic development disparities in the EU is tested and validated based on the working hypotheses in Table 11 and connected to possible directions and policies for sustainable development of the green economy, including based on the convergence of the SDG-Green Deal, represents the real benefits that this research brings to common policies.

## 6. Conclusions

The scientific approach taken to quantify the evolution of structural changes in the economy under the impact of the EU green policies and the quality of life represents a point of confirmation of the importance of involving all Member States in this European strategic approach. The syncope or destructured implementation of the EU objectives in this field can be a significant barrier to reaching the targets proposed by the leading EU forums in the medium and long term.

The research identified progress made in the field from the literature study. This is in line with research objective O1: To identify in the literature the main concepts, trends and approaches related to sustainable development in the context of large-scale introduction of R&D in economic activity.

This scientific approach involved the study of indicators specific to the monitoring of the Green Deal objectives and the consolidation of a database in accordance with the purpose of the research, which allowed to achieve the O2 objective of the research: the construction of a consolidated database of the evolution of the main monitoring indicators of the results obtained in the implementation of the Green Deal at European level and at the level of the selected Member States.

During the definition and testing of the model, the main characteristics and vulnerabilities of Green Deal implementation in the Member States were observed, which was the subject of objectives O3 (Definition of the econometric model to quantify development for the green economy at EU and national levels) and O4 (Testing, validation of the model and proposals for practical solutions and policies to accelerate Green Deal development).

The obtained results confirm the correctness of the hypotheses assumed by the authors of this paper, namely that economic development is a factor in implementing strategies through R & D and also the need to develop a program to train developing countries in this activity with definite benefits on successful implementation of the green economy. The proposed model by us is validated both by the consistency of the analysed data (20 years) and by the possibility of implementation at the level of each EU member state.

The novelty of the study lies in the realisation of a bi-directional approach (SDG-Green Deal) with regional consolidation of policy developments and the highlighting of development disparities at the green policy level through a valid econometric model, the results of which allow supranational decision-makers to approach green policy development from a regional perspective.

In conclusion, based on the regression analysis, differences in the development of the green economy were found mainly due to differences in funding in the field. As a common element, the green economy and dedicated policies require significant financial investments, which are difficult for developing the Member States to achieve.

The limits of the model consist of the relatively small number of indicators and the authors considering certain aspects of the green economy.

We mention that there are no conflicts of interest.

## 7. Future Work

The authors aim to extend the model to other branches of the green economy, seeking to identify better traceability of sustainable development models in the EU.

## Figures and Tables

**Table 1:** The model of analysis for the EU.

Model Summary ^b^						
Model	R	R Square	Adjusted R Square	Std. Error of the Estimate	Change Statistics	
					R Square Change	F Change
1	0.994 ^a^	0.988	0.983	0.02084	0.988	192.176
Change Statistics	df1	df2	Sig. F Change	Durbin-Watson		
1	6	14	0	1.955		

^a^ Predictors: (Constant), *r &dpersEU*, *oxiEU*, *greenhouseEU*, *phosrivEU*, *wasteEU*, *renewEU*. ^b^ Dependent Variable: *r&dEU*. Source: Data operated under IBM-SPSS 25 IBM, Armonk, NY, USA).

**Table 2:** ANOVA method for the EU.

ANOVA ^a^						
Model		Sum of Squares	df	Mean Square	F	Sig.
1	Regression	0.501	6	0.083	192.176	0.000 ^b^
	Residual	0.006	14	0		
	Total	0.507	20			

^a^ Dependent Variable: *r&dEU*. ^b^ Predictors: (Constant), *r&dpersEU*, *oxiEU*, *greenhouseEU*, *phosrivEU*, *wasteEU*, *renewEU*. Source: Data operated under IBM-SPSS 25 (IBM, Armonk, NY, USA).

**Table 3:** The model of analysis for Bulgaria during 2000–2020.

Model Summary ^b^						
Model	R	R Square	Adjusted R Square	Std. Error of the Estimate	Change Statistics	
					R Square Change	F Change
1	0.928 ^a^	0.86	0.801	0.07512	0.86	14.379
Change Statistics	df1	df2	Sig. F Change	Durbin-Watson		
1	6	14	0	1.787		

^a^ Predictors: (Constant), *r&dpersBG*, *greenhouseBG*, *phosrivBG*, *oxiBG*, *wasteBG*, *renewBG*. ^b^ Dependent Variable: *r&Dbg*. Source: Data operated under IBM-SPSS 25 (IBM, Armonk, NY, USA).

**Table 4:** ANOVA method for Bulgaria.

ANOVA ^a^						
Model		Sum of Squares	df	Mean Square	F	Sig.
1	Regression	0.487	6	0.081	14,379	0.000 ^b^
	Residual	0.079	14	0.006		
	Total	0.566	20			

^a^ Dependent Variable: *r&dBG*. ^b^ Predictors: (Constant), *r&dpersBG*, *greenhouseBG*, *phosrivBG*, *oxiBG*, *wasteBG*, *renewBG*. Source: Data operated under IBM-SPSS 25 (IBM, Armonk, NY, USA).

**Table 5:** The model of analysis for Ireland.

Model Summary ^b^						
Model	R	R Square	Adjusted R Square	Std. Error of the Estimate	Change Statistics	
					R Square Change	F Change
1	0.870 ^a^	0.757	0.653	0.14616	0.757	7.275
Change Statistics	df1	df2	Sig. F Change	Durbin-Watson		
1	6	14	0.001	1.496		

^a^ Predictors: (Constant), *r&dpersIR*, *wasteIR*, *oxiIR*, *greenhouseIR*, *phosrivIR*, *renewIR*. ^b^ Dependent Variable: *r&dIR*. Source: Data operated under IBM-SPSS 25 (IBM, Armonk, NY, USA).

**Table 6:** ANOVA method for Ireland.

ANOVA ^a^						
Model		Sum of Squares	df	Mean Square	F	Sig.
1	Regression	0.932	6	0.155	7.275	0.001 ^b^
	Residual	0.299	14	0.021		
	Total	1.231	20			

^a^ Dependent Variable: *r&dIR*. ^b^ Predictors: (Constant), *r&dpersIR*, *wasteIR*, *oxiIR*, *greenhouseIR*, *phosrivIR*, *renewIR*. Source: Data operated under IBM-SPSS 25 (IBM, Armonk, NY, USA).

**Table 7:** The model of analysis for France.

Model Summary ^b^						
Model	R	R Square	Adjusted R Square	Std. Error of the Estimate	Change Statistics	
					R Square Change	F Change
1	0.841 ^a^	0.706	0.581	0.04739	0.706	5.616
Change Statistics	df1	df2	Sig. F Change	Durbin-Watson		
1	6	14	0.004	1.200		

^a^ Predictors: (Constant), *r&dpersF*, *greenhouseF*, *phosrivF*, *oxiF*, *renewF*, *wasteF*. ^b^ Dependent Variable: *r&dF*. Source: Data operated under IBM-SPSS 25 (IBM, Armonk, NY, USA).

**Table 8:** ANOVA method for France.

ANOVA ^a^						
Model		Sum of Squares	df	Mean Square	F	Sig.
1	Regression	0.076	6	0.013	5.616	0.004 ^b^
	Residual	0.031	14	0.002		
	Total	0.107	20			

^a^ Dependent Variable: *r&dF*. ^b^ Predictors: (Constant), *r&dpersF*, *greenhouseF*, *phosrivF*, *oxiF*, *renewF*, *wasteF.* Source: Data operated under IBM-SPSS 25 (IBM, Armonk, NY, USA).

**Table 9:** The model of analysis for Romania.

Model Summary ^b^						
Model	R	R Square	Adjusted R Square	Std. Error of the Estimate	Change Statistics	
					R Square Change	F Change
1	0.731 ^a^	0.535	0.336	0.04464	0.535	2.684
Model	Change Statistics					
	df1	df2	Sig. F Change	Durbin-Watson		
1	6	14	0.06	1.271		

^a^ Predictors: (Constant). *r&dpersRO*, *phosrivRO*, *greenhouseRO*, *oxiRO*, *wasteRO*, *renewRO*. ^b^ Dependent Variable: *r&dRO*. Source: Data operated under IBM-SPSS 25 (IBM, Armonk, NY, USA).

**Table 10:** ANOVA method for Romania.

ANOVA ^a^						
Model		Sum of Squares	df	Mean Square	F	Sig.
1	Regression	0.032	6	0.005	2.684	0.060 ^b^
	Residual	0.028	14	0.002		
	Total	0.06	20			

^a^ Dependent Variable: *r&dRO*. ^b^ Predictors: (Constant). *r&dpersRO*, *phosrivRO*, *greenhouseRO*, *oxiRO*, *wasteRO*, *renewRO*. Source: Data operated under IBM-SPSS 25 (IBM, Armonk, NY, USA).

**Table 11:** Connecting research hypotheses with policy suggestions.

Hypotheses	Observation	Policies
**H1:***The economic development is a booster of R&D activity and an indirect instrument by which the objectives of the green economy and quality of life pursued at EU level through specific policies and strategies, can be more easily achieved.* This is supported by research carried out by Kim et al. [15], Cling et al. [19], Wang et al. [16] and Junwei Ma et al. [29].	At European level, the main syncope in supporting the strategic objectives on green policies and quality of life is reflected during the economic crisis The changes compared with the general profile are significant indicates the weak adherence to the sustainable development objectives considered by the EU.	Increasing expenditure on implementing R&D results in economic activity. Targeting of R&D expenditure should be done on the basis of SDG-Green Deal convergence.
**H2:***There are significant differences between the general European model and the national models, which demonstrates that the European proposed desideratum regarding the sustainable development involves national disruptions when they are transferred through financing programs across the Member States’ economies.* This is supported by research carried out by Yuan et al. [17], Liu and Dong [27], Chai et al. [31].	There are significant differences in the impact of syncopes in terms of strategies at the national level. The effect of the Markov chains on the national economies after the manifestation of syncopes in the general profile is noticeable. The syncopes last at least a few years after the manifestation at the general profile or take over elements of cyclicality.	Mitigating regional socio-economic disparities allows better implementation of sustainable economic development. The efficient use of European funds is an active tool to implement the Green Deal concept at regional level.
**H3:***The Member States with higher economic development always tend to approach faster and more faithfully to the European model than those Member States with a developing economy.* This is supported by research by Yuan et al. [17], Odugbesan et al. [28], Chai et al. [31], Zhang et al. [32].	Comparative analysis between the analysed economic entities—values closest to the general profile are obtained at the average level for developed countries (France) while the gap for the developing countries is higher.	From a policy point of view, the most effective tools are legislative ones. Thus for developing countries there is a need to accelerate legislative change and public administration reform. Another vulnerability is corruption, which needs to be reduced through strong retaliatory policies.
**H4:***The structural differences between the implementation of the sustainable development objectives across the Member States represent economic decelerators for the implementation of the policies at European level.* This is supported by research carried out by Li and Gan [26], Liu and Dong [27], Junwei Ma et al. [29], Qian et al. [30], Chai et al. [31], Zhang et al. [32].	The significant structural differences observed during the modelling allowed the definition of differentiated Member State profiles. In implementing sustainable development, the uneven level of economic dislocation affects the achievement of Green Deal objectives.	Monitoring vulnerabilities regarding the absorption of European funds. Implementing smart management in public administration.

## Data Availability

Not applicable.
